# Using Plant Functional Traits to Define the Biomass Energy Potential of Invasive Alien Plant Species

**DOI:** 10.3390/plants12183198

**Published:** 2023-09-07

**Authors:** Alex Ceriani, Michele Dalle Fratte, Gustavo Agosto, Antonio Montagnoli, Bruno Enrico Leone Cerabolini

**Affiliations:** Department of Biotechnology and Life Sciences, University of Insubria, Via Dunant 3, 21100 Varese, Italy; gustavo.agosto@uninsubria.it (G.A.); antonio.montagnoli@uninsubria.it (A.M.); bruno.cerabolini@uninsubria.it (B.E.L.C.)

**Keywords:** bioeconomy, biofuels, eradication, global change, global spectrum of plant form and function, Grime’s CSR plant strategies, plant functional traits, restoration ecology

## Abstract

The eradication of invasive alien plant species (IAPS) is mandatory worldwide, but the resulting biomass is still considered waste. The energy use of biomasses obtained from IAPS eradication may represent ecological and economic benefits, creating synergies with restoration projects. We evaluated whether the growth forms and functional types identified using the functional space of 63 IAPS corresponded to a possible bioenergy use through multivariate analysis techniques. We extracted leaf and nutrient traits and Grime’s CSR plant strategies from an existing database. We calculated the carbon-to-nitrogen ratio (C:N) and gross heating value (GHV) as indicators of biochemical or thermal processes, respectively. For 10 species, we measured the above-ground biomass C:N and GHV (including leaves, stems and branches) and correlated them with those of leaves and with plant adaptive strategies. We identified four groups of IAPS indicative of the main trade-offs between plant economics and size variation, which respectively correlated with C:N and GHV. Herbaceous IAPS were better suited to biochemical processes, and woody IAPS to thermal ones. Overall, Grime’s CSR strategies were the best tool to define the IAPS bioenergy potential. In the long term, competitive and ruderal IAPSs can represent a reusable feedstock until their complete eradication.

## 1. Introduction

Biological invasion has increased with globalization [1] and is considered the second major cause of global biodiversity loss [2]. Plant species become naturalized when introduced in areas with suitable ecological and climatic conditions outside their natural distribution range, overcoming abiotic and biotic barriers to survival [3]. Only naturalized plants with the potential to spread over large areas, i.e., those that produce reproductive offspring at considerable distances from the parent, can be defined as invasive alien plant species (IAPS) [4]. Despite occupying the same functional space as native species [5], many IAPS have higher growth rates and larger sizes than native species and are likely to allocate more aboveground biomass [6]. In addition, IAPS may find more favorable habitats due to rapid climate change [7], thus becoming increasingly dominant in the host environment.

Since IAPS eradication is mandatory worldwide (e.g., EU Regulation 1143/2014; US Executive Order 13751/2016), they represent significant economic expenses, with worldwide mean annual costs reaching USD 162.7 billion [8]. After eradication, the resulting biomass is generally considered as waste to be disposed of, although it may be turned into feedstock for bioenergy production [9], fully embracing circular economy principles that are essential for achieving the goals of international agreements for countering global climate change (e.g., United Nations Development Goals, European Green Deal). Given IAPS’ diversity in growth forms and functional attributes [10], it is presumable that different energy uses (i.e., processes for energy production, such as: anaerobic digestion, biofermentation, combustion, pyrolysis) can be made of their biomass. Thus, it is relevant to identify fast and easy tools that could help detect a priori the best use of IAPS biomass as feedstock. A starting point to develop such tools could be plant functional traits that determine the global-scale plant functional space [11].

Trade-offs among plant functional traits have been summarized in the global spectrum of plant form and functions (GSPFF), representing plant size and economics [11], whose variations also mirror Grime’s triangle theory of competitor, stress tolerator, and ruderal (CSR) plant strategies [12]. Although more traits are needed to identify the GSPFF, CSR strategies can be calculated based on three easily identifiable leaf traits (LDMC, LA, SLA) [13]. The plant functional space could predict the potential bioenergy use of IAPS biomass, further supporting the investment of resources in regeneration after disturbance. According to Grime [12], acquisitive and fast-growing IAPS with large organ sizes, i.e., competitive (C) IAPS, produce in a short time large amounts of biomass. At the same time, acquisitive and fast-growing IAPS with small organ sizes, i.e., ruderals (R), are associated with faster regeneration. Thus, IAPS with these growing and reproductive characteristics can be indicative of possible repeated use for energy production throughout the years. However, to our knowledge, this link between plant functional space and IAPS bioenergy use has not yet been evaluated.

Biochemical (anaerobic digestion and biofermentation) [14] and thermal (combustion and pyrolysis) [15] processes are promising techniques for processing IAPS biomass. The biochemical processes involve microorganisms. In anaerobic digestion, they convert organic matter into methane-rich gas [16]. In biofermentation, they convert biomass cellulose and hemicellulose into bioethanol [14]. Thermal processes require high temperatures. Combustion utilizes fuel that is burned to produce energy [17], while pyrolysis involves combustion with little or no oxygen, resulting in gaseous, liquid, and solid (biochar) products [18]. The affinity of biomass may be determined by two stoichiometric parameters measured on plant biomass, respectively, the carbon-to-nitrogen ratio (C:N) [16] and gross heating value (GHV) [18]. C:N determines microbial activity during anaerobic digestion and biofermentation [16], while GHV represents the heat released during complete biomass combustion [14]. Considering that plant functional traits help classify bioenergy crops in terms of productivity and energy use [19], it might be expected that the affinity of IAPS biomass for biochemical or thermal processes could be explored by grouping them according to growth forms or plant functional types (i.e., sets of species sharing similar responses to the environment) inferred by plant functional space, although these are determined using mainly leaf traits.

In this study, based on a dataset of 63 IAPS representative of the administrative regions’ blacklists of the southern-central Alps (Table S1), (i) we evaluated whether the growth forms (i.e., woody and herbs) and functional types inferred by the plant functional space could predict the best biomass energy use of IAPS, and (ii) we determined, based on a subset of 10 most widespread IAPS, whether plant adaptive strategies evident within the plant functional space are representative of aboveground biomass indicators of biochemical and/or thermal processes (respectively, C:N and GHV). This research could greatly assist conservation policies, by identifying potential revenues from IAPS eradication in invaded sites within the study area. Furthermore, the conceptual framework that we propose relies on easily measurable plant functional traits and thus can serve as a straightforward tool for determining the best ways to use other IAPS biomass within the study area as well as globally.

## 2. Results

### 2.1. Principal Component Analysis

**Figure 1:**
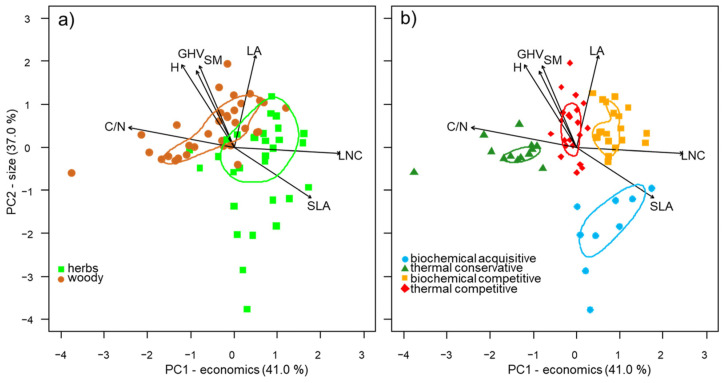
Principal component analysis of trait values of the 63 IAPS selected in this study grouped by (**a**) growth form and (**b**) bioenergy use types according to the cluster they belong to (see Figure 2). Lines represent the 50th percentile of the distribution. Legend: C:N = carbon-to-nitrogen ratio, GHV = gross heating value, H = plant height, LA = leaf area, LNC = leaf nitrogen content SLA = specific leaf area, SM = seed mass.

The first two principal components of the PCA (PC1 and PC2) explained 78% (respectively, 41% and 37%) of the dataset’s total variance (Figure 1a), encompassing the variation from conservative to acquisitive leaves, i.e., the leaf economics spectrum (PC1-economics), and the increase in plants and organ size (PC2-size).

SLA and LNC were positively correlated with PC1-economics, and SM, H, and LA with PC2-size (Figure 1a, Table 1). Considering the bioenergy use indicators, C:N was negatively correlated with PC1-economics, while GHV was positively correlated with PC2-size (Figure 1a, Table 1).

**Figure 2:**
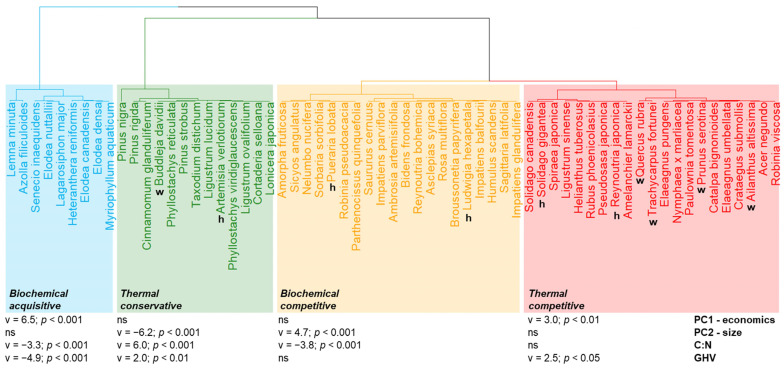
Dendrogram resulting from hierarchical clustering on the principal components (PC1-economics and PC2-size) of the invasive alien plant species (IAPS) present in the Lombardy region. Both axes contributed significantly (*p* < 0.001) to the clustering at k = 4, Eta^2^ = 0.70 and 0.77, respectively. The value of the v.test (v) and its significance (*p*) in the contributions to each cluster by PC1-economics, PC2-size, and by the indicators of biochemical and thermal processes (C:N and GHV) are also reported in the figure. Accordingly, classes can be grouped into biochemical-acquisitive, thermal-conservative, biochemical-competitive, and thermal-competitive. We indicated the five herbaceous (h) and five woody (w) IAPS with the highest ecological impact in Lombardy selected for the analysis of the aboveground biomass (see Table S1).

**Figure 3:**
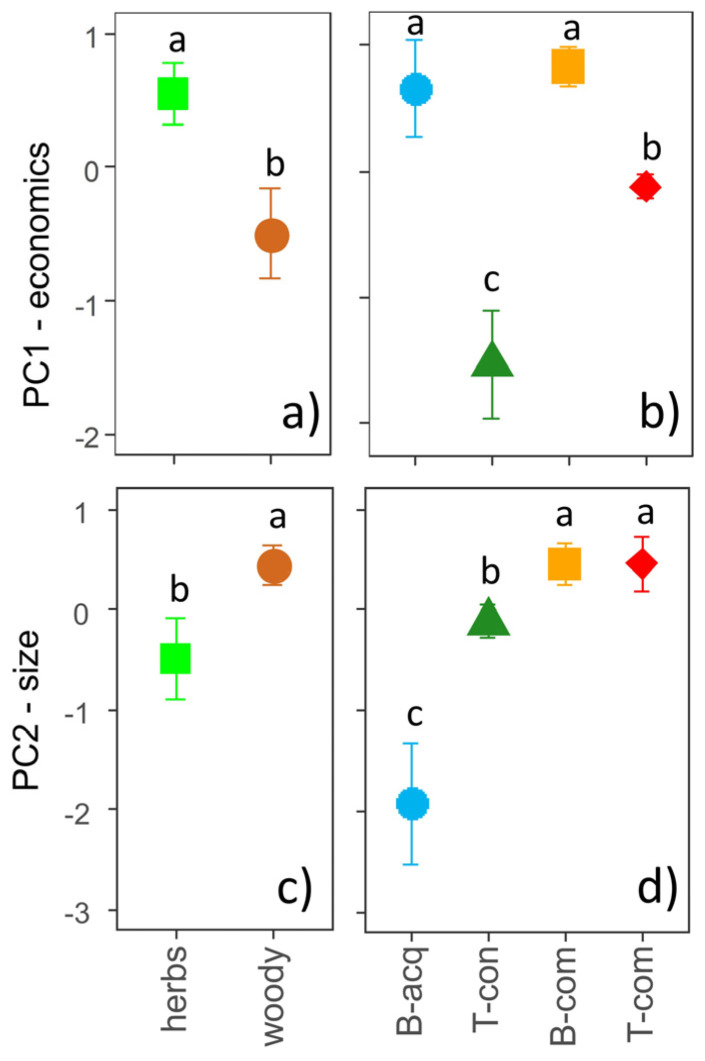
Mean values (±standard error × 1.96) of the scores of the two axes of the principal component analysis (PC1-economics and PC2-size) for different growth forms (**a**,**c**) and bioenergy use types (**b**,**d**). Results of the ANOVA are reported in each subplot; small letters indicate post hoc comparisons (*p* < 0.05). Legend: B-acq = biochemical acquisitive, B-com = biochemical competitive, T-con = thermal conservative, T-com = thermal competitive.

Both PC1-economics (eta^2^ = 0.70, *p* < 0.001) and PC2-size (eta^2^ = 0.77, *p* < 0.001) contributed to the identification of four potential bioenergy use types (Figure 1b and Figure 2): (i) biochemical-acquisitive (*n* = 9), positively associated with PC1-economics and negatively with C:N and GHV; (ii) thermal-conservative (*n* = 13), negatively associated with PC2-size and positively with C:N and GHV; (iii) biochemical-competitive (*n* = 20), positively associated with PC2-size and negatively with C:N; (iv) thermal-competitive (*n* = 21), positively associated with PC1-economics and GHV. Biochemical-acquisitive and competitive IAPS had higher PC1-economics values than thermal-conservative and competitive ones (Figure 3b). Biochemical- and thermal-competitive IAPS exhibited higher PC2-size values than thermal-conservative IAPS and even more so versus biochemical-acquisitive ones (Figure 3d).

Similarly, the growth forms showed a specific pattern in the PCA multidimensional space (F = 20.2, *p* < 0.001; Figure 1a). Herbaceous IAPS had significantly higher PC1-economics values than woody ones, which displayed significantly higher PC2-size values (Figure 3a,c).

### 2.2. CSR Plant Strategies

**Figure 4:**
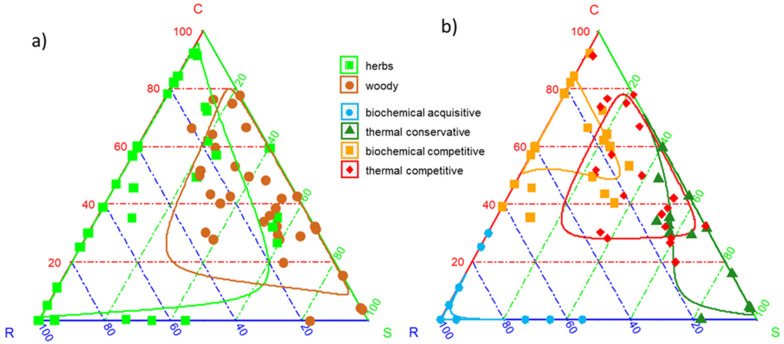
Ternary visualization of Grime’s competitive, stress-tolerant, ruderal (CSR) plant strategies of the 63 invasive alien plant species (IAPS) selected in this study, grouped by (**a**) growth form and (**b**) bioenergy use types according to the cluster they belong to (see Figure 2). Lines represent the 50th percentile of the distribution.

Leaf C:N and GHV were negatively correlated with R-strategy and positively with S-strategy, but did not correlate with C-strategy (Table 1). Although the IAPS spread evenly in most of the CSR ternary space, they displaced differently according to growth forms and bioenergy use types (Figure 4a,b). 

**Figure 5:**
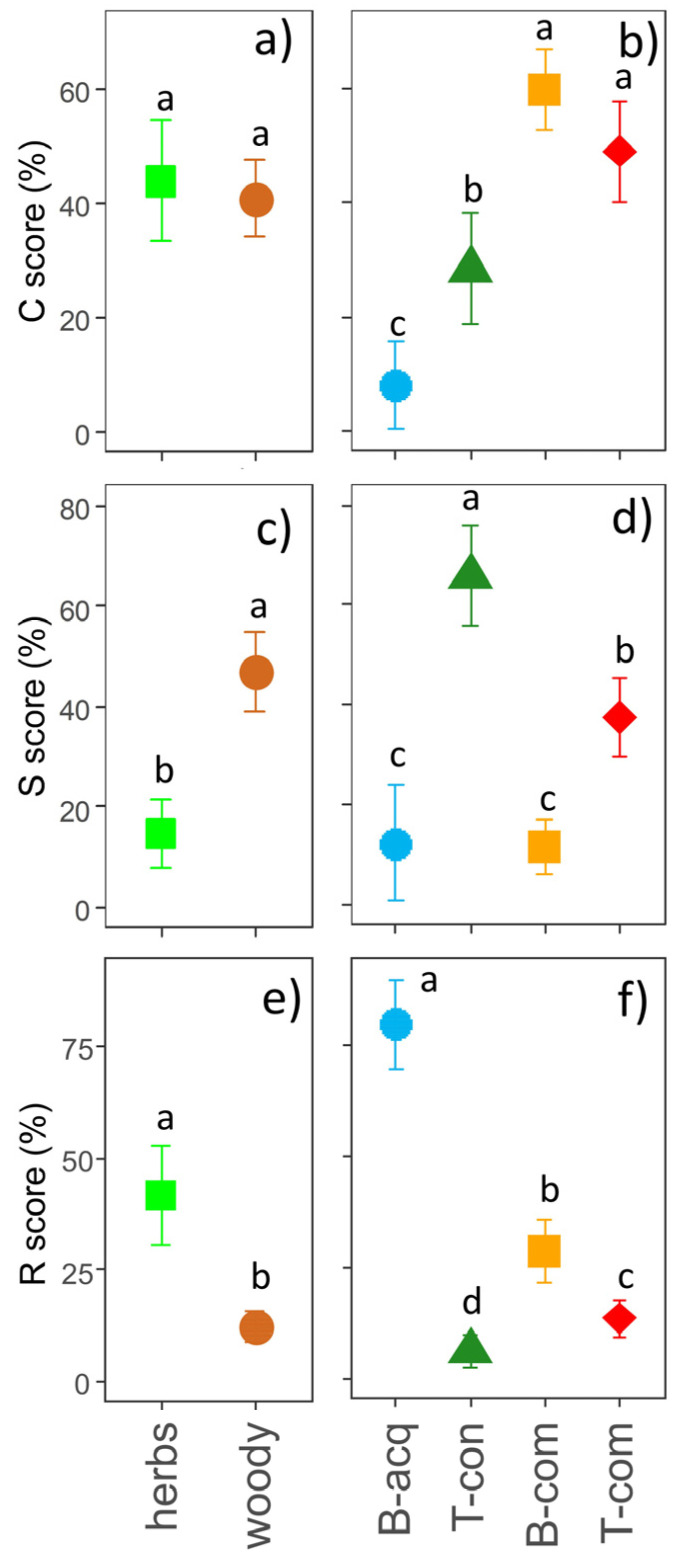
Mean values (±standard error × 1.96) of the competitive (C), stress-tolerant (S), and ruderal (R) strategy scores for different growth forms (**a**,**c**,**e**) and bioenergy use types (**b**,**d**,**f**). Results of the ANOVA are reported in each subplot; small letters indicate pos hoc comparisons (*p* < 0.05). Legend: B-acq = biochemical acquisitive, B-com = biochemical competitive, T-con = thermal con-servative, T-com = thermal competitive.

Herbaceous and woody IAPS had similar C-scores (Figure 5a), while woody IAPS displayed significantly higher S-scores (Figure 5c), and herbaceous IAPS had significantly higher R-scores (Figure 5e). Biochemical- and thermal-competitive IAPS exhibited the highest C-scores, thermal-conservative IAPS had intermediate C-scores, and biochemical-acquisitive IAPS had the lowest C-scores (Figure 5b). Thermal-conservative IAPS exhibited the highest S-scores, thermal-competitive IAPS intermediate S-scores, and biochemical-acquisitive and competitive IAPS the lowest S-scores (Figure 5d). Finally, biochemical-acquisitive and thermal-conservative IAPS showed the highest and lowest R-scores, respectively (Figure 5f), and biochemical- and thermal-competitive IAPS had intermediate R-scores (Figure 5f).

### 2.3. Carbon-to-Nitrogen Ratio and Gross Heating Value

**Figure 6:**
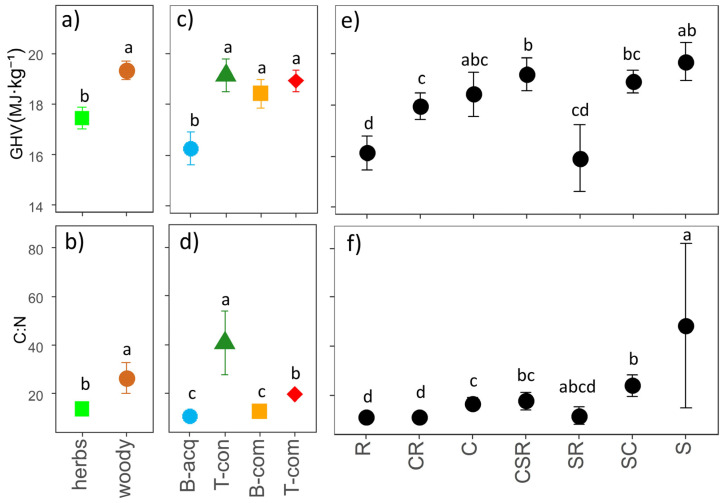
Mean values (±standard error × 1.96) of the gross heating value (GHV, above) and carbon-to-nitrogen ratio (C:N, below) scores for different growth forms (**a**,**b**), bioenergy use types (**c**,**d**), and CSR (competitive, stress-tolerant, ruderal) plant strategy types (**e**,**f**). Results of the ANOVA are reported in each subplot; small letters indicate post hoc comparisons (*p* < 0.05). Legend: B-acq = biochemical acquisitive, B-com = biochemical competitive, T-con = thermal con-servative, T-com = thermal competitive.

Considering GHV and C:N, we found significant differences between different growth forms (Figure 6a,b), bioenergy use types (Figure 6c,d), and CSR strategy categories (Figure 6e,f). Woody IAPS showed higher C:N and GHV than herbaceous ones (Figure 6a,b). Biochemical-acquisitive IAPS displayed significantly lower GHV compared to the three other bioenergy use types, which were similar to each other (Figure 6c). Biochemical-acquisitive and competitive IAPS exhibited the lowest C:N, while thermal-conservative and competitive IAPS had the highest and intermediate C:N values, respectively (Figure 6d). Among the CSR strategy categories, stress-tolerant (S) and C–S–R strategist (CSR) IAPS had the highest GHV, while ruderal (R), competitive–ruderal (CR) and stress-tolerant–ruderal (SR) IAPS had the lowest, which were similar to each other (Figure 6e). Also, competitive (C) and stress-tolerant–competitive (SC) IAPS displayed intermediate GHV, which was significantly higher only compared to ruderal (R) ones (Figure 6e). The C:N of the stress-tolerant (S) IAPS was significantly higher than that of all other categories (Figure 6f). In contrast, ruderal (R) and competitive–ruderal (CR) IAPS showed the lowest C:N (Figure 6f). All of the other categories had intermediate C:N values, with stress-tolerant–competitive (SC) IAPS showing significantly higher values compared to competitive (C) IAPS, and C–S–R strategist (CSR) IAPS showing values similar to those of both of the two previous categories (Figure 6f). Only stress-tolerant–ruderal (SR) IAPS did not show significant differences compared to all other categories (Figure 6f).

### 2.4. Aboveground Biomass C:N and GHV Relationships with Leaf Traits and Plant Strategies

**Figure 7:**
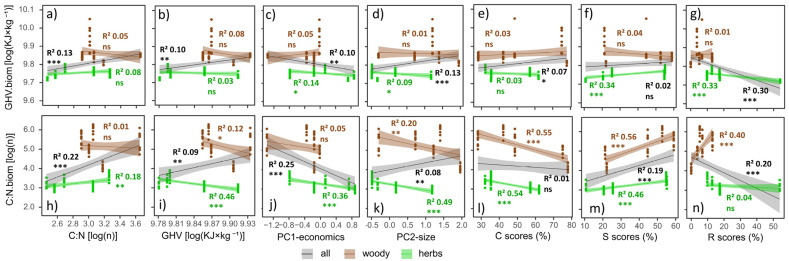
Linear regression between aboveground biomass C:N (**h**–**n**), GHV (**a**–**g**) and PC1-economics (**c**,**j**), PC2-size (**d**,**k**), C- (**e**,**j**), S- (**f**,**m**), and R- (**g**,**n**) strategy axes, and C:N (**a**,**h**) and GHV (**b**,**i**) leaf values considering the 10 selected species (all) and grouped by growth forms (herbs and woody). Legend: C:N = carbon-to-nitrogen ratio, GHV = gross heating value, PC1-economics = first principal component, PC2-size = second principal component, C = degree of competitiveness, S = degree of stress tolerance, R = degree of ruderality. The values of the linear regression coefficients are reported: determination coefficient, R^2^, and level of significance, *p* (ns = not significant, * = *p* ≤ 0.05, ** = *p* ≤ 0.01, *** = *p* ≤ 0.001).

Independently of the growth forms of the 10 selected IAPS (Figure 2, Table S1), the biomass GHV showed a significant positive linear relationship with leaf C:N and GHV, PC2-size, and C-strategy, the latter with a low R^2^, and a negative linear relationship with PC1-economics and R-strategy (Figure 7a–g). No relationships were detected between biomass GHV and S-strategy (Figure 7f). The biomass C:N showed a significant positive linear relationship with leaf C:N and GHV, PC2-size, and S-strategy, and a negative relationship with PC1-economics and R-strategy (Figure 7h–n). We did not observe a significant relationship between the biomass C:N and the C-strategy (Figure 7l).

Significant linear relationships existed when analyzing herbaceous and woody IAPS separately, even if the patterns were slightly different. The biomass GHV of herbaceous IAPS exhibited a negative linear relationship with the R-strategy (Figure 7g) and, less significantly, with PC1-economics and PC2-size (Figure 7c,d), and a positive linear relationship with S-strategy (Figure 7f). No relationships with C:N, GHV, and C-strategy were observed (Figure 7a,b,e). The biomass GHV of the woody IAPS did not show significant linear relationships with the traits considered (Figure 7a–g). The biomass C:N of herbaceous IAPS had a positive linear relationship with C:N and S-strategy (Figure 7h,m), and a negative one with all other traits (Figure 7i–l), except for the R-strategy (Figure 7n). The biomass C:N of woody IAPS displayed a positive linear relationship with both S- and R-strategy (Figure 7m,n), and a negative relationship with GHV, PC2-size, and C-strategy (Figure 7i,k,l). No relationships were detected between the biomass C:N of woody IAPS and leaf C:N and PC1-economics (Figure 7h,j).

## 3. Discussion

The 63 examined IAPS showed distinct coordination between the leaf economics spectrum (PC1-economics) and the plant and organ size dimension (PC2-size), mirroring the GSPFF [11], and spread evenly throughout the CSR ternary space, suggesting that they can overlap the functional space of native plant species [5,20]. Therefore, successful invasion is linked to traits that allow a higher ability to capture and retain resources [21,22,23], which in turn are also desirable as optimal bioenergy feedstock [19,24], supporting the rationale of using the biomass of eradicated IAPS for energy purposes. The bioenergy use indicators (C:N and GHV) showed a distinct pattern within the GSPFF. C:N mirrored the variation of the plant economics spectrum [25], i.e., from fast (low C:N) to slow-growing species (high C:N), indicating the relative investment in structure (carbon) and cell functioning (nitrogen) [26]. C:N denotes biomass affinity to biochemical processes, as it determines microorganisms’ ability to perform anaerobic digestion and biofermentation [16]. GHV reflected the variation from small (low GHV) to large (high GHV) plants and organ size [11] and was also related to the SR-strategy variation, being linked to wood density and higher carbon and lignin content [27,28], the latter being a hindrance to producing biofuels (e.g., [29]).

Our findings pointed out that the growth forms and plant functional types inferred by plant functional space can be used as easy and fast tools to predict the potential bioenergy use of IAPS, which was our first objective. Herbaceous IAPS showed more acquisitive/ruderal characteristics, contrary to woody ones, which exhibited a more conservative/stress-tolerant nature [30]. As expected, herbaceous IAPS were more suitable for biochemical processes (low C:N), while woody IAPS were better suited for thermal ones (high GHV) (e.g., [17]). Additionally, we did not observe any differences in C-strategy between herbaceous and woody IAPS [22], thus suggesting that both growth forms can produce significant amounts of aboveground biomass, a desirable feedstock property, depending on nutritional value. We classified the IAPS into four bioenergy use types based on their affinity for C:N, GHV, and their identity within the GSPFF: two were more related to biochemical processes, and two to thermal ones. IAPS with higher affinity for biochemical processes had C:N close to optimal values for anaerobic digestion and biofermentation [31] and were more acquisitive, corresponding to the CR-strategy variation [13]. However, IAPS destined for thermal processes were associated with higher C:N and GHV and were more conservative, resembling the CS-strategy variation [13].

We further identified two subtypes (i.e., acquisitive and competitive) within biochemical IAPS, and two others (i.e., conservative and competitive) within thermal IAPS, based on their differences along PC1-economics and PC2-size. Biochemical-acquisitive IAPS could be identified as ruderals, i.e., IAPS with a shorter lifespan and low structural investments, contrary to thermal-conservative IAPS, identifiable as stress tolerators [12]. Biochemical and thermal-competitive IAPS were more related to the C-selected syndrome, including rapid growth and higher biomass production [12]. Interestingly, herbaceous and woody IAPS were distributed among all bioenergy use types, except for biochemical-acquisitive IAPS, which were all hydrophytes, except *Senecio inaequidens*, confirming that hydrophytes are mostly R-selected [32]. These IAPS have high water and nitrogen content, supporting their suitability for biochemical processes (e.g., [33]). Only two hydrophytes, i.e., *Nelumbo nucifera* and *Nymphae x marliacea*, were respectively clustered with the biochemical- and thermal-competitive IAPS, supporting the C-strategy selection of *Nymphaeiden* species [5]. Conversely, the thermal-conservative IAPS mainly corresponded to woody species, except *Artemisia verlotiorum* and *Cortaderia selloana*, whose high carbon contents are related to oil and secondary metabolites or silicophytoliths, respectively (e.g., [34,35]). The two competitive types differed according to their C:N values and in line with evidences in the literature: e.g., *Solidago gigantea* and *Reynoutria japonica* have already been tested for thermal processes, and *Impatiens glandulifera* and *Humulus scandens* for biochemical processes [15,36].

Considering the 10 IAPS most widespread in the Lombardy region (Table S1), the correlations between plant adaptive strategies and biomass indicators of bioenergy use broadly mirrored those identified by the same indicators referred to leaf only. Thus, according to our second objective, we may assert that plant adaptive strategies give information about the potential IAPS bioenergy use, as also supported by positive correlations between indicators calculated using leaves and biomass data. However, C:N was positively related to the PC2-size, and GHV to the C-strategy scores only when calculated on biomass data, likely due to the higher carbon content associated with stem density and plant size of woody IAPS [11]. In contrast, GHV was not related to the S-strategy scores when calculated on biomass data, perhaps due to the high variability of the GHV of *Ailanthus altissima* (Table S2), linked to the different chemical composition of the stem elements [37]. Furthermore, different patterns emerged within each growth form, likely due to stem nutrient contents, but further data are necessary to deepen this understanding. Although using only 10 IAPS may seem limiting, this is a preliminary validation that plant trait analysis can serve as a quick tool for identifying the best end-use of IAPS biomass. Such analyses can be expensive and time-consuming, making this method a valuable alternative.

Comparing IAPS destined for biochemical or thermal processes, they are also differentiated by the fast–slow economics spectrum, which is representative of the relative growth rate [25,38], and thus of IAPS regrowth speed after management interventions [39]. Similarly, in the CSR space, stress-tolerant IAPS tend to have slow growth, attaining long life spans [12], and it would take a long time to recolonize and produce enough biomass after eradication. In contrast, competitive and ruderal IAPS can have a higher ability to spread and invade after removal [22], especially considering the high reproductive investment these species have [13], which contributes to their expansion and presence on non-native territory even after eradication efforts. This means that ongoing management strategies are necessary for these species, which thus represent a reusable feedstock until their complete eradication allowing an economic return [15]. Managing the IAPS population holistically by using biomass from management in a circular economy framework can reconcile biodiversity goals with climate change mitigation [40]. Crucially, IAPS biomass, as a waste from eradication and management measures, is readily available and present in large quantities (e.g., [9,40]). Hence, for bioenergy use, their biomass would probably be less expensive than other, traditional biomasses and might avoid problems related to the sustainability of feedstock production for bioenergy, representing a solution to solve the “food, energy, environment trilemma” [41] and boosting restoration activities.

## 4. Materials and Methods

### 4.1. Dataset

We selected 63 IAPS (30 herbaceous and 33 woody; Table S1) on the blacklist (regional law n.10/2008 and DGR n.XI/2658) of the Lombardy administrative region (Northern Italy), as an example for the southern-central Alps. For each IAPS, we extracted leaf traits data (plant height, H; leaf area, LA; leaf carbon content, LCC; leaf dry matter content, LDMC; leaf hydrogen content, LHC; leaf nitrogen content, LNC; specific leaf area, SLA) and Grime’s C-, S-, and R-strategy scores from the FIFTH [42] and LIFTH [26] databases. Accordingly, IAPS were also classified into seven categories comprising primary and secondary strategies [12]: C (competitive), CR (competitive–ruderal), R (ruderal), SR (stress-tolerant–ruderal), S (stress-tolerant), SC (stress-tolerant–competitive), CSR (C–S–R strategist). Further methodological details are reported by Dalle Fratte et al. [26]. Seed mass (SM) was derived from the Seed Information Database (https://ser-sid.org/, accessed on 4 September 2023). Missing SM data (21 IAPS) were obtained using the “Gap Filling” function of the “BHPMF” R-package [43]. To this end, we used all of those species with SM values from the authors’ dataset described above (*n* = 239) to include a greater number of taxa, allowing better prediction accuracy.

For each IAPS, we calculated the C-to-N ratio (C:N) and the gross heating value (GHV) using the formula [28]:

GHV = 3.55 × C^2^ – 232 × C – 2230 × H + 51.2 × C × H + 131 × N + 20,600

where C, H and N indicate the percentage of leaf carbon, hydrogen, and nitrogen content, respectively.

We then selected the five herbaceous and five woody IAPS most widespread in the Lombardy region (Table S1) based on their occurrence in grid cells of 10 × 10 km^2^ [44]. For each of these IAPS, during the 2022 growing season at the peak biomass of the different species, we sampled the aboveground biomass (including leaves, stems, branches, and reproductive portions when already present) of 10 replicates (or six in the case of *Ludwigia hexapetala*) from highly invaded sites located at similar altitudes and at a great distance (100 m) from the roadsides or from other potential anthropic disturbances that could impact the biomass nutrient content. The biomass was chipped (~7 cm^2^) using a woodchipper (GeoTech PCS70L) and first dried in an aerated room for 10 days at 25 °C. Subsamples were then collected and dried at 105 °C for 24 h. Dry biomass was mixed and ground, and three randomly selected sub-replicates were processed with a CHNS analyzer (FlashEA 1112 series Thermo Fisher Scientific, Rodano, Italy) to measure aboveground biomass C, H, and N content, which were used to calculate the biomass C:N and GHV according to the formula reported above. The species nomenclature follows the classification of Galasso et al. [45].

### 4.2. Data Analysis

All statistical analyses were computed with R software [46]. We performed a principal component analysis (PCA), followed by varimax rotation, on functional traits data using the “principal” function in the package “psych” [47]. We selected traits relevant to the GSPFF (LA, SLA, LNC, SM, H; [11]) and C:N and GHV as indicative of the affinity of biomasses for, respectively, biochemical (anaerobic digestion and bio-fermentation; [16,31]) and thermal processes (combustion and pyrolysis; [18]). All of the trait data were log-transformed, centered, and scaled before running the PCA. After determining the significant components of the PCA, we applied hierarchical clustering on the principal components, using the function “HCPC” of the package “FactoMineR” [48] to check the presence of bioenergy use types within the GSPFF. We used the package “ggtern” [49] to visualize the ternary CSR diagram.

We then tested for differences in the spatial arrangement of growth forms (herbaceous vs. woody) and bioenergy use types within the multidimensional space determined by the significant components of the PCA and within the CSR ternary space through distance-based multivariate ANOVA [50] with 9999 random permutations of trait values among species. To this end, we used the function “adonis” of the package “vegan” [51], based on Euclidean or Bray–Curtis distance for the PCA multidimensional space or the CSR ternary space, respectively. We used the Bray–Curtis dissimilarity to account for the constant sum constraint in multivariate analysis among compositional data such as the CSR ones [52]. We tested for pairwise differences in the values of the significant PCA components and each C-, S-, and R-strategy axis among the growth forms and bioenergy use types using standard univariate ANOVA with 9999 random permutations and the Benjamini and Hochberg adjustment method. We used the same method to compare the C:N and GHV values among growth forms, bioenergy use types, and the seven categories of CSR strategies.

We then built linear models with 9999 permutations using the “lmp” function of the package “lmPerm” [53] to check for linear relations between C:N and GHV calculated on the aboveground biomass and the values of significant components of the PCA, C-, S-, and R- strategy, and leaf C:N and GHV. Before running these models, the data were log-transformed.

## Figures and Tables

**Table 1:** Pearson’s correlation coefficients among plant functional traits, the proportion of C-, S- and R-selection, and the first two axes of the principal component analysis for the 63 selected IAPS.

	LNC	SLA	H	LA	GHV	SM	C	S	R	PC1-economics	PC2-size
**C:N**	−0.99 ***	−0.70 ***	−0.66 ***	0.0 ns	0.48 ***	0.42 ***	−0.12 ns	0.75 ***	−0.63 ***	−0.97 ***	0.18 ns
**LNC**			−0.48 ***	0.07 ns	−0.32 **	−0.35 **	0.18 ns	−0.73 ***	0.56 ***	0.97 ***	−0.06 ns
**SLA**			−0.66 ***	−0.30 *	−0.49 ***	−0.47 ***	−0.22 ns	−0.67 ***	0.88 ***	0.71 ***	−0.46 ***
**H**				0.46 ***	0.62 ***	0.73 ***	0.23 ns	0.53 ***	−0.74 ***	−0.48 ***	0.76 ***
**LA**					0.41 ***	0.44 ***	0.91 ***	−0.29 *	−0.56 ***	0.20 ns	0.84 ***
**GHV**						0.51 ***	0.18 ns	0.43 ***	−0.60 ***	−0.34 **	0.70 ***
**SM**							0.30 *	0.32 **	−0.60 ***	−0.31 *	0.75 ***
**C**								−0.47 ***	−0.47 ***	0.31 *	0.66 ***
**S**									−0.56 ***	−0.83 ***	0.06 ns
**R**										0.54 ***	−0.69 ***

C:N = carbon to nitrogen ratio, LNC = leaf nitrogen content, SLA = specific leaf area, H = plant height, LA = leaf area, GHV = gross heating value, SM = seed mass, C, S, R = degree of competition, stress tolerance, ruderality, ns = not significant, * = *p* ≤ 0.05, ** = *p* ≤ 0.01, *** = *p* ≤ 0.001.

## Data Availability

The complete data collected in the research are available from the authors.

## Supplementary Materials

The following supporting information can be downloaded at: https://www.mdpi.com/article/10.3390/plants12183198/s1, Table S1: List of the 63 IAPS and trait data selected for this study, Table S2: Mean and standard error of the aboveground biomass indicators of the 10 selected IAPS.
