# Supplementary material for: Using Plant Functional Traits to Define the Biomass Energy Potential of Invasive Alien Plant Species
Source: Plants (Basel). 2023 Sep 7;12(18):3198. doi: 10.3390/plants12183198 (PMC10535227; doi:10.3390/plants12183198)
Supplement: Supplementary file 1 [file plants-12-03198-s001.zip › plants-2581813-supplementary.pdf]

plants

Supplementary materials

Using the plant functional traits to define the biomass energy potential of invasive alien plant species

Alex Ceriani [1,*], Michele Dalle Fratte [1,*], Gustavo Agosto [1], Antonio Montagnoli [1], Bruno Enrico Leone Cerabolini [1]

[1] Department of biotechnology and life sciences, University of Insubria, via Dunant 3, 21100, Varese, Italy
* Correspondence: aceriani3@uninsubria.it (AC); michele.dallefratte@gmail.com (MDF)

Table S1: List of the 63 IAPS and traits data selected for this study (the 10 IAPS selected for biomass analysis are highlighted). Legend: Neotrop. = Neotropical, (x) = commercial hybrid, Tcomp = thermal competitive, Tcons = thermal conservative, Bacqu = Biochemical acquisitive, Bcomp = Biochemical competitive.

Species	Range of distribution	growth form	bioenergy use type	C:N	GHV (KJ Kg^{-1})	LA (mm^2)	SLA (mm^2 mg^{-1})	LDMC (%)	LNC (%)	H (mm)	SM (mg)	PC1-economics	PC2-size	C (%)	S (%)	R (%)
Acer negundo	America North	woody	Tcomp	19.8	19506.6	8241.4	27.0	25.4	2.5	12500	28.0	0.0	0.9	50.3	23.7	25.9
Ailanthus altissima	Asia	woody	Tcomp	20.2	19315.8	64503.0	12.9	35.5	2.4	10000	29.2	0.0	1.2	75.0	21.2	3.7
Ambrosia artemisiifolia	America North	herbs	Bcomp	11.8	18446.0	3802	20	22	3.8	766	3.6	0.7	0.1	49.5	23.2	27.2
Amelanchier lamarckii	America North	woody	Tcomp	22.7	18417.7	2900.5	36.3	34.3	2.0	5500	18.3	-0.3	0.2	28.1	38.8	33.1
Amorpha fruticosa	America North	woody	Bcomp	9.8	20200.8	5235.7	25.2	31.0	5.0	2500	7.8	1.2	0.9	40.4	34.8	24.8
Artemisia verlotiorum	Asia East	herbs	Tcons	26.1	18022.0	2761.5	13.2	36.5	1.7	1250	1.1	-0.8	-0.5	35.4	54.8	9.8
Asclepias syriaca	America North	herbs	Bcomp	13.9	17291.4	16255.3	15.6	21.7	3.1	1250	6.2	0.5	0.2	72.5	15.0	12.5
Azolla filiculoides	America Neotr.	herbs	Bacqu	10.0	15246.2	0.9	41.4	29.5	3.5	55	0.1	0.2	-2.9	0.0	44.6	55.4
Bidens frondosa	America North	herbs	Bcomp	13.5	19248.0	3465.1	36.4	17.8	3.5	1620	4.9	0.7	0.3	45.6	6.0	48.4
Broussonetia papyrifera	Asia East	woody	Bcomp	13.6	17936.4	12770.9	21.0	25.8	3.2	3500	2.1	0.6	0.4	59.9	23.7	16.4
Buddleja davidii	Asia	woody	Tcons	38.5	20625.2	4268.7	11.9	30.2	1.3	2500	0.1	-1.4	-0.3	48.6	44.6	6.8
Catalpa bignonioides	America North	woody	Tcomp	20.3	18319.2	23200.3	12.1	33.2	2.3	6500	24.4	-0.2	0.7	66.8	29.5	3.7
Cinnamomum glanduliferum	Asia	woody	Tcons	56.6	19172.2	2667.5	8.5	47.5	0.9	11250	172.0	-2.1	0.3	31.5	68.5	0.0
Cortaderia selloana	America South	herbs	Tcons	28.7	17844.5	10117.2	6.1	33.5	1.6	1750	4.7	-1.0	-0.1	59.6	40.4	0.0
Crataegus submollis	America North	woody	Tcomp	23.4	19924.3	6156.1	21.9	32.4	2.1	6000	84.7	-0.3	0.8	42.9	36.5	20.6
Elaeagnus pungens	Asia East	woody	Tcomp	17.0	20286.7	2488.4	13.5	33.1	2.9	2000	16.0	0.0	0.6	36.4	51.7	11.8
Elaeagnus umbellata	Asia	woody	Tcomp	18.7	19644.5	1974.5	8.1	40.0	2.6	6000	19.3	-0.4	0.6	32.3	67.7	0.0
Elodea canadensis	America North	herbs	Bacqu	8.7	16301.7	26.3	76.4	17.5	4.5	325	0.0	1.0	-1.8	0.2	4.7	95.1
Elodea densa	America South	herbs	Bacqu	8.5	17623.9	104.0	92.6	14.1	5.1	500	0.0	1.3	-1.2	5.1	0.0	94.9
Elodea nuttallii	America North	herbs	Bacqu	11.4	15859.7	27.7	62.3	22.5	3.3	375	0.0	0.4	-2.0	0.3	26.4	73.3
Helianthus tuberosus	America North	herbs	Tcomp	21.5	16847.4	10553.1	14.2	20.2	1.9	1500	3.8	-0.4	-0.2	73.7	13.8	12.5
Heteranthera reniformis	America Neotr.	herbs	Bacqu	7.5	18011.8	1542.7	64.4	10.6	5.8	115	0.0	1.7	-0.9	30.0	0.0	70.0
Humulus scandens	Asia West	herbs	Bcomp	10.1	16463.8	17677.2	18.9	16.5	4.0	2000	18.0	1.0	0.4	82.2	0.0	17.8
Impatiens balfourii	Asia	herbs	Bcomp	13.4	17223.7	4661.9	50.4	10.0	3.2	920	3.7	0.7	-0.2	47.0	0.0	53.0
Impatiens glandulifera	Asia	herbs	Bcomp	11.2	16959.6	8776.6	45.4	11.6	3.7	1500	9.8	1.1	0.2	57.0	0.0	43.0
Impatiens parviflora	Asia East	herbs	Bcomp	9.2	18760.3	4549.2	94.4	10.1	5.0	728	8.0	1.6	0.3	39.1	0.0	60.9

Lagarosiphon major	Africa	herbs	Bacqu	13.6	16582.1	17.6	46.2	24.4	3.0	350	0.0	0.1	-2.0	0.0	34.4	65.6
Lemna minuta	tropical (Euroasia, Africa, America)	herbs	Bacqu	13.1	15121.9	2.4	155.5	10.1	2.7	2	0.0	0.3	-3.8	0.0	0.0	100.0
Ligustrum lucidum	Asia East	woody	Tcons	34.8	20295.6	3841.3	7.2	37.6	1.4	5500	31.0	-1.3	0.5	42.8	57.2	0.0
Ligustrum ovalifolium	Asia East	woody	Tcons	28.5	19662.4	1301.7	13.4	29.0	1.7	1500	21.0	-0.9	0.0	32.6	55.8	11.6
Ligustrum sinense	Asia East	woody	Tcomp	22.6	20328.7	667.1	14.3	34.0	2.2	4500	17.0	-0.6	0.3	20.0	64.3	15.7
Lonicera japonica	Asia East	woody	Tcons	31.8	20418.5	2413.5	15.0	33.9	1.6	3500	1.8	-1.0	0.0	34.1	51.4	14.6
Ludwigia hexapetala	America	herbs	Bcomp	11.7	17693.5	1324.3	28.9	18.4	3.7	487.5	2.9	0.7	-0.3	35.3	10.7	53.9
Myriophyllum aquaticum	America South	herbs	Bacqu	12.6	16010.3	455.1	203.2	6.8	3.0	500	1.2	0.9	-1.2	11.2	0.0	88.8
Nelumbo nucifera	tropical (Africa and Asia)	herbs	Bcomp	13.8	17970.5	324706.8	17.2	17.3	3.2	1500	1.8	0.9	0.8	92.3	1.0	6.7
Nymphaea marliacea	Eurasia and America (x)	herbs	Tcomp	18.9	18136.5	43936.7	13.9	17.4	2.4	1100	2.5	0.1	0.3	91.4	2.5	6.1
Parthenocissus quinquefolia	America North	woody	Bcomp	15.3	20314.5	21116.3	26.4	22.3	3.3	3500	25.2	0.6	1.1	66.4	13.3	20.3
Paulownia tomentosa	Asia East	woody	Tcomp	17.0	18406.1	47907.0	17.4	25.8	2.7	8000	0.2	0.3	0.4	76.6	14.6	8.9
Phyllostachys reticulata	Asia West	woody	Tcons	33.1	17033.9	3191.6	12.4	54.3	1.3	9000	7.6	-1.3	-0.3	29.2	64.9	5.9
Phyllostachys viridiglaucescens	Asia East	woody	Tcons	31.1	17685.1	3024.5	15.8	45.9	1.4	9000	4.8	-1.1	-0.2	29.8	57.5	12.6
Pinus nigra	Europe (North-East)	woody	Tcons	116.1	20823.1	167.3	5.4	21.4	0.5	8000	26.3	-3.8	-0.6	4.5	95.5	0.0
Pinus rigida	America North	woody	Tcons	40.6	19144.5	456.6	5.2	40.3	1.2	14250	8.0	-2.0	-0.1	15.7	84.3	0.0
Pinus strobus	America North	woody	Tcons	33.1	18844.6	78.5	8.9	47.9	1.4	19000	17.0	-1.7	-0.3	3.7	96.3	0.0
Prunus serotina	America North	woody	Tcomp	18.3	19038.0	2079.8	15.4	40.7	2.6	8500	100.6	-0.2	0.7	28.2	58.9	12.9
Pseudosasa japonica	Asia West	woody	Tcomp	19.8	17474.4	6698.5	14.6	49.6	2.2	3500	4.0	-0.3	0.0	38.6	52.5	8.9
Pueraria lobata	Asia East	herbs	Bcomp	13.0	20205.6	26015.6	26.8	29.0	3.8	4500	12.3	0.9	1.2	62.0	20.5	17.5
Quercus rubra	America North	woody	Tcomp	21.3	19232.7	6309.9	12.1	44.2	2.3	17500	3143.0	-0.4	1.4	42.2	52.6	5.2
Reynoutria bohemica	Asia East (x)	herbs	Bcomp	16.6	17595.8	35492.7	31.7	16.6	2.6	1750	2.5	0.5	0.2	78.3	0.0	21.7
Reynoutria japonica	Asia West	herbs	Tcomp	21.2	19091.7	9609.9	18.7	25.9	2.3	1800	2.1	-0.2	0.2	57.2	25.5	17.4
Robinia pseudoacacia	America North	woody	Bcomp	12.6	19101.8	11289.3	17.8	32.6	3.7	8500	19.2	0.7	1.0	53.2	33.1	13.7
Robinia viscosa	America North	woody	Tcomp	16.4	19062.5	9818.8	16.1	36.3	2.9	18000	25.5	0.2	1.0	49.8	38.8	11.4
Rosa multiflora	Asia East	woody	Bcomp	15.0	18667.1	7690.0	30.1	31.1	3.1	1500	8.4	0.5	0.3	43.6	30.2	26.2
Rubus phoenicolasius	Asia East	woody	Tcomp	20.8	18002.7	9970.6	27.7	26.7	2.2	2500	3.3	-0.1	0.0	51.7	23.3	25.0
Sagittaria latifolia	America North	herbs	Bcomp	11.6	17136.2	28176.2	19.5	11.1	3.6	600	0.6	0.9	0.0	84.5	0.0	15.5
Saururus cernuus	America North	herbs	Bcomp	8.8	17927.0	13295.5	53.2	11.0	4.9	600	0.7	1.6	0.1	60.0	0.0	40.0
Senecio inaequidens	Africa	herbs	Bacqu	13.3	15863.5	156.3	16.6	15.2	2.9	630	0.3	0.0	-1.4	25.3	0.0	74.7
Sicyos angulatus	America North	herbs	Bcomp	13.8	17792.0	20152.5	80.6	10.0	3.2	3500	102.2	1.0	0.7	59.6	0.0	40.4
Solidago canadensis	America North	herbs	Tcomp	15.8	18158.0	1292.7	14.1	34.8	2.8	1150	0.1	0.0	-0.6	26.6	59.2	14.1
Solidago gigantea	America North	herbs	Tcomp	14.7	17939.2	1553.7	13.8	31.1	3.0	1024	0.2	0.1	-0.5	32.2	55.1	12.8
Sorbaria sorbifolia	Asia	woody	Bcomp	18.8	21454.9	36192.6	26.5	31.6	2.8	2000	50.3	0.4	1.3	64.2	20.5	15.3
Spiraea japonica	Asia West	woody	Tcomp	19.3	20358.9	3052.5	35.7	31.4	2.6	1000	0.0	0.1	-0.4	30.3	35.4	34.4
Taxodium distichum	America North	woody	Tcons	30.4	19261.9	23.3	14.1	38.0	1.6	20000	87.0	-1.5	-0.2	0.0	82.4	17.6
Trachycarpus fortunei	Asia East	woody	Tcomp	24.0	20050.8	776815.9	6.4	44.6	2.2	8500	181.3	-0.2	2.0	77.9	22.1	0.0

Table S2: Mean and standard error (se) of the aboveground biomass indicators (C:N and GHV) calculated on leaves and entire aboveground biomass and PC1-economics, PC2-size, C, S and R of the ten selected IAPS.

Species	CN.biomass		GHV.biomass (KJ Kg^{-1})		CN.leaves	GHV.leaves (KJ Kg-1)	PC1-economics	PC2-size	C (%)	S (%)	R (%)
	**mean**	**se**	**mean**	**se**	**mean**	**mean**	**mean**	**mean**	**mean**	**mean**	**mean**
Ailanthus altissima	145.6	9.2	20888.8	417.7	20.2	19315.8	0.0	1.2	75.0	21.2	3.7
Artemisia verlotiorum	37.2	2.4	17177.4	88.4	26.1	18022.0	-0.8	-0.5	35.4	54.8	9.8
Buddleja davidii	231.7	37.6	18971.6	46.1	38.5	20625.2	-1.4	-0.3	48.6	44.6	6.8
Ludwigia hexapetala	25.6	2.1	16644.7	45.1	11.7	17693.5	0.7	-0.3	35.3	10.7	53.9
Prunus serotina	271.5	26.8	18891.0	86.5	18.3	19038.0	-0.2	0.7	28.2	58.9	12.9
Pueraria lobata	19.3	0.8	16956.9	61.5	13.0	20205.6	0.9	1.2	62.0	20.5	17.5
Quercus rubra	412.0	39.1	18824.7	136.4	21.3	19232.7	-0.4	1.4	42.2	52.6	5.2
Reynoutria japonica	20.9	1.1	17417.9	73.8	21.2	19091.7	-0.2	0.2	57.2	25.5	17.4
Solidago gigantea	29.8	1.7	17747.3	80.3	14.7	17939.2	0.1	-0.5	32.2	55.1	12.8
Trachycarpus fortunei	61.1	3.6	18258.5	56.7	24.0	20050.8	-0.2	2.0	77.9	22.1	0.0
